# Regulation of central carbon metabolism and storage polymers by PTS^Ntr^ in *Rhizobium leguminosarum*

**DOI:** 10.1128/aem.01073-26

**Published:** 2026-09-30

**Authors:** Olivia Tjahjono, Jingkai Wang, Evan Turner, Jürgen Prell, Wei E. Huang, Philip S. Poole, Carmen Sánchez-Cañizares

**Affiliations:** 1 Department of Biology, University of Oxford 6396, Oxford, United Kingdom; 2 Department of Engineering Science, University of Oxford 6396, Oxford, United Kingdom; 3 Soil Ecology, Rheinisch-Westfälische Technische Hochschule, Aachen, Germany; 4 Instituto de Recursos Naturales y Agrobiología de Salamanca (IRNASA), Consejo Superior de Investigaciones Científicas (CSIC) 16379, Salamanca, Spain; The University of Tennessee Knoxville, Knoxville, Tennessee, USA

**Keywords:** symbiosis, plant-microbe interactions, bacterial metabolism, metabolic regulation, storage polymers, phosphotransferase system, nitrogen fixation, rhizobium, proteobacteria, carbon metaboilsm

## Abstract

The coordination of intracellular carbon and nitrogen levels is essential for optimal bacterial growth and, in rhizobia, for survival in the soil, root colonization, and symbiotic interactions with their host plants. Here, we show that the phosphotransferase system PTS^Ntr^, a global regulator of carbon and nitrogen metabolism, modulates the tricarboxylic acid (TCA) cycle activity and the accumulation of three major carbon storage polymers: glycogen, polyhydroxybutyrate, and exopolysaccharide in *Rhizobium leguminosarum*. The unphosphorylated form of the effector protein ManX is sufficient for the full activation of the TCA cycle dehydrogenase enzymes. Accordingly, loss of *manX* reduced dehydrogenase activity and redirected overflow carbon into storage polymers, phenocopying wild-type cells grown under nitrogen starvation. We further demonstrate that carbon metabolism in *R. leguminosarum* is also tightly regulated by the HPr kinase (HprK) protein. In an *hprK* mutant, loss of NPr Ser48 phosphorylation favors phosphotransfer through the histidyl arm and is predicted to increase phosphorylation of downstream PTS^Ntr^ components. When grown on glucose, the *hprK* mutant accumulates elevated intracellular pyrimidine levels and exhibits increased malate dehydrogenase and TCA cycle activity, consistent with a misregulated shift toward gluconeogenesis. This mutant also overproduces exopolysaccharide, an effect potentially mediated by crosstalk between phosphorylated PtsN and the ChvI/ChvG two-component regulatory system. Together, these data suggest that HprK exerts pleiotropic control over processes regulated by PTS^Ntr^, influencing central metabolism while repressing exopolysaccharide production, likely by promoting the unphosphorylated state of PtsN.

**IMPORTANCE:**

Bacteria must continuously balance growth, nutrient availability, and storage to ensure fitness and survival in fluctuating environments. However, how regulatory networks integrate central metabolism with carbon storage remains poorly understood. This study reinforces the role of the PTS^Ntr^ system as a central coordinator of carbon routing and polymer accumulation. By revealing how ManX and HprK-dependent phosphorylation influence central metabolism and the production of major carbon storage polymers in *Rhizobium leguminosarum*, we show that disruption of PTS^Ntr^ signaling reprograms carbon allocation, uncoupling growth from carbon storage and mimicking nitrogen starvation even under nitrogen-replete conditions. These findings highlight a regulatory link between carbon-nitrogen signaling and bacterial storage strategies, with implications for cellular survival and plant symbiosis. More broadly, our work illustrates how global metabolic regulators shape bacterial physiological states, providing new insights into mechanisms that support microbial adaptation in complex environments.

## INTRODUCTION

Biological nitrogen fixation (BNF) is essential for sustainable agriculture by supplying bioavailable nitrogen and reducing the reliance on synthetic fertilizers (1, 2). Symbiotic rhizobia that carry out this process persist in soil as free-living bacteria and, in legume hosts forming indeterminate nodules, as intracellular growth-arrested bacteroids (3, 4). Each stage of this complex life cycle is intricately linked to cellular energy homeostasis and to carbon and nitrogen metabolism (5, 6). Tight regulation of bacterial carbon and nitrogen metabolism is therefore essential to support growth, meet energy demands, and enable adaptation to diverse environments and across different lifestyles.

Among the major systems coordinating nitrogen and carbon metabolism in Alphaproteobacteria (α-proteobacteria) are the Ntr/PII pathway and the nitrogen-related phosphotransferase system PTS^Ntr^. The Ntr/PII system mediates both post-translational and transcriptional effects in response to nitrogen availability, which is sensed by PII proteins through the glutamine/α-ketoglutarate ratio (7–11). As a key tricarboxylic acid (TCA) cycle intermediate, α-ketoglutarate (also known as 2-oxoglutarate) is converted to glutamine through the GS-GOGAT (glutamine synthetase-glutamate synthase) pathway (11). Thus, this ratio signals the relative state of both carbon and nitrogen metabolism. A low glutamine/α-ketoglutarate ratio signals nitrogen deficiency, leading to upregulation of nitrogen and amino acid uptake in free-living bacteria (12, 13). In contrast, a high glutamine/α-ketoglutarate ratio signals nitrogen sufficiency.

PTS^Ntr^ is primarily a post-translational signaling system that regulates various cell functions via protein-protein interactions (14–17). It is a three-step phosphorelay cascade in which PtsP (EI^Ntr^) autophosphorylates using phosphoenolpyruvate (PEP) as the phosphate donor transfers the phosphate to NPr (PtsO) and ultimately to the output regulators PtsN (EIIA^Ntr^) and ManX (EIIA^Man^), whose phosphorylation states determine the different downstream regulatory targets. PtsP has a GAF sensing domain that detects changes in intracellular nitrogen availability, with autophosphorylation being inhibited by glutamine-binding in Alphaproteobacteria (17). Unphosphorylated ManX has been shown to be necessary for full activation of the TCA cycle (17–19), while phosphorylated PtsN activates ATP-binding cassette (ABC) transporters and interacts with the two-component regulatory system ChvI/ChvG controlling exopolysaccharide (EPS) biosynthesis (17, 20, 21).

In Alphaproteobacteria, PTS^Ntr^ is additionally modulated by a bifunctional HPr kinase/phosphorylase (HprK/P) (19, 22). This enzyme phosphorylates NPr at a conserved serine (Ser) residue, which in turn reduces subsequent histidine phosphorylation of NPr by PtsP. Although homologous to the Firmicutes HprK/P, which phosphorylates HPr at Ser46 rather than NPr, the Alphaproteobacterial HprK/P lacks the N-terminal phosphate-binding domain characteristic of the Firmicutes protein (22–24). Evidence for HprK/P activity in Alphaproteobacteria comes from *Brucella melitensis*, where this enzyme phosphorylates NPr on Ser61 in an ATP-dependent reaction. The kinase is inhibited by inorganic phosphate and stimulated by fructose-1,6-bisphosphate (FBP) (19). In *Sinorhizobium meliloti*, deletion of *hprK* causes a severe growth defect and significantly enhances succinate-mediated catabolite repression (22). Similarly, our previous work in *Rhizobium leguminosarum* showed that the *hprK* mutant displays severe growth defects, further supporting a role for HprK as a component of this integrated PTS^Ntr^ system involved in the regulation of both nitrogen and carbon metabolism (17).

**Fig 1:**
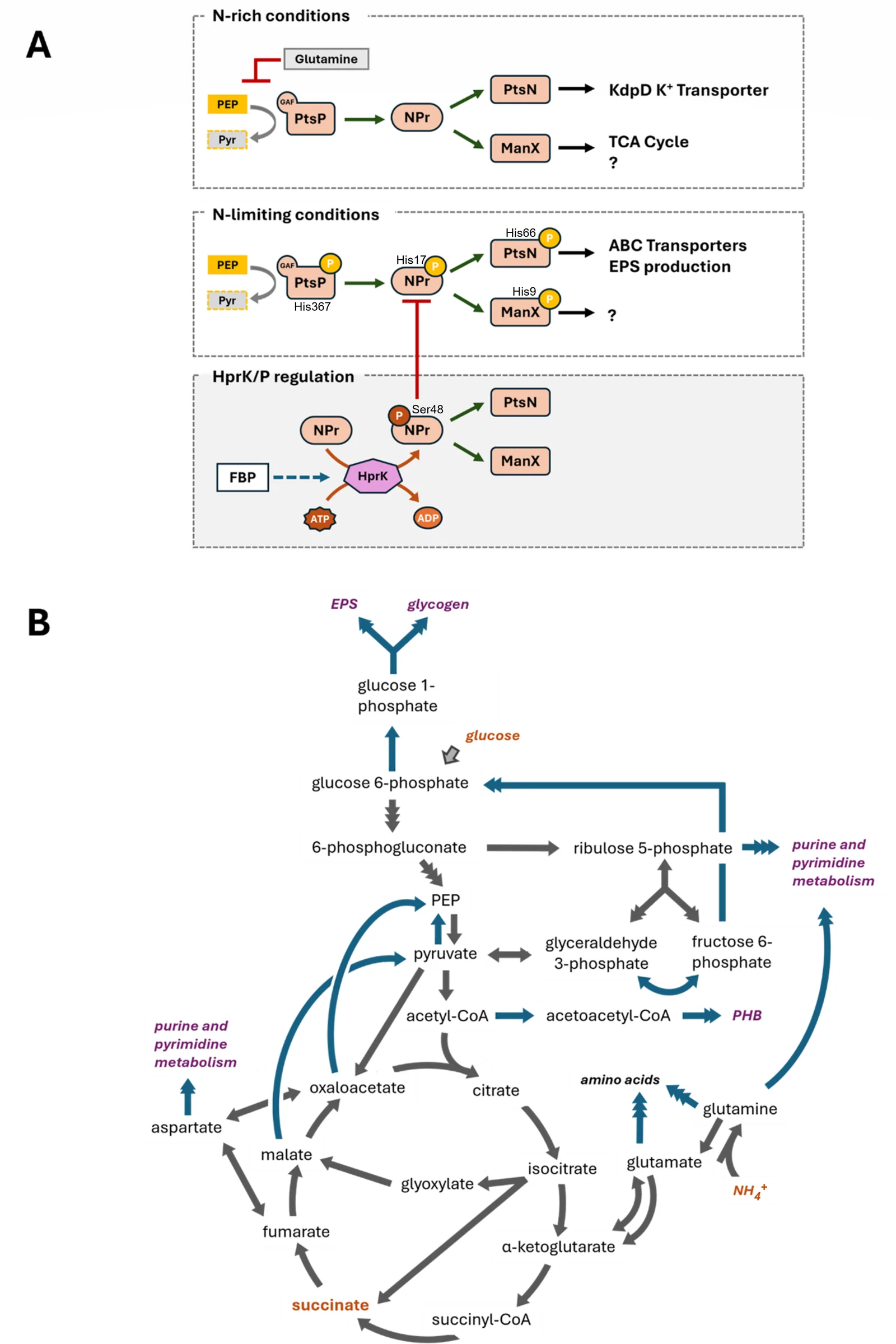
PTS^Ntr^ regulatory system and carbon metabolism in *R. leguminosarum*. (**A**) Schematic diagram of the current understanding of PTS^Ntr^ regulation in Rlv3841. Under N-rich conditions, glutamine inhibits PtsP autophosphorylation. In contrast, under N-limiting conditions, autophosphorylated PtsP transfers its phosphate to NPr on His17 and downstream effectors PtsN and ManX. The phosphorylation state of NPr is additionally modulated by an alternative HprK-dependent serine phosphorylation at Ser48. Yellow circles indicate histidine phosphorylation, while red circles indicate serine phosphorylation. FPB: fructose-1,6-bisphosphate. (**B**) Overview of the carbon metabolism pathways in Rlv3841. Blue arrows indicate anabolic metabolic pathways. Orange labels are key carbon sources (glucose and succinate) and the nitrogen source (ammonia) used in this study, while purple labels indicate polymers (EPS, glycogen and PHB) and other compounds (pyrimidines) quantified in this work. Selected reactions are shown based on previous models (25, 26).

In addition to the *hprK* mutant, other PTS^Ntr^ mutants such as *ptsP*, *npr*, and *manX* exhibited reduced growth, highlighting the importance of this regulatory system for cellular fitness (17). An overview of the PTS^Ntr^ regulatory interactions and associated cellular functions known to date in the reference strain *R. leguminosarum* bv. viciae 3841 (Rlv3841) is provided in Fig. 1A, while key features of carbon metabolism relevant to this study are summarized in Fig. 1B. Importantly, direct interactions between ManX and key metabolic enzymes have been shown in only two bacterial species: in *B. melitensis*, yeast 2-hybrid (Y2H) assays showed an interaction between ManX and SucA, a component of the α-ketoglutarate dehydrogenase (α-KGDH) complex (19), whereas in *Pseudomonas putida*, PtsN was shown to bind pyruvate dehydrogenase (PDH) by co-immunoprecipitation and mass spectrometry (18). However, similar interactions with central metabolic enzymes have not been demonstrated in other species, including *R. leguminosarum*.

A link between PTS^Ntr^ and internal carbon storage has been proposed, but the underlying regulatory mechanisms are not yet understood (17, 20–22, 27–30). In *S. meliloti*, the promoter region of *manX* contains putative consensus binding sites for the polyhydroxybutyrate (PHB)-binding transcriptional regulator PhaR, and its expression increased under PHB-producing growth conditions (30). Additionally, polyhydroxyalkanoates accumulate in the *ptsN* mutant of *P. putida* (28), while phosphorylated PtsN (PtsN~P) was shown to interact with the ChvI/ChvG two-component system in *R. leguminosarum* to regulate the synthesis of the extracellular carbon-polymer exopolysaccharide (EPS) (17). This function is conserved in *S. meliloti*, where PtsN interacts with the homologous system ChvI/ExoS to modulate the production of the surface polymers galactoglucan and succinoglycan (20, 21). Additionally, as shown in *S. meliloti* (22, 29) and more recently in *R. leguminosarum* (17), the *hprK* mutant also accumulates EPS when grown on nitrogen-excess media.

In this work, we investigate how PTS^Ntr^ contributes to the allocation of carbon between central metabolism and storage in *R. leguminosarum*. Using biochemical assays complemented by Raman spectroscopy, we characterize patterns of intracellular and extracellular polymer accumulation in wild-type cells under contrasting nitrogen conditions. We then assess the physiological consequences of disrupting the PTS^Ntr^ components ManX, PtsN, and HprK by integrating new measurements of central metabolic enzyme activities and carbon polymer accumulation with previously characterized growth phenotypes.

## RESULTS

### ManX modulates the activity of TCA cycle dehydrogenases

**Fig 2:**
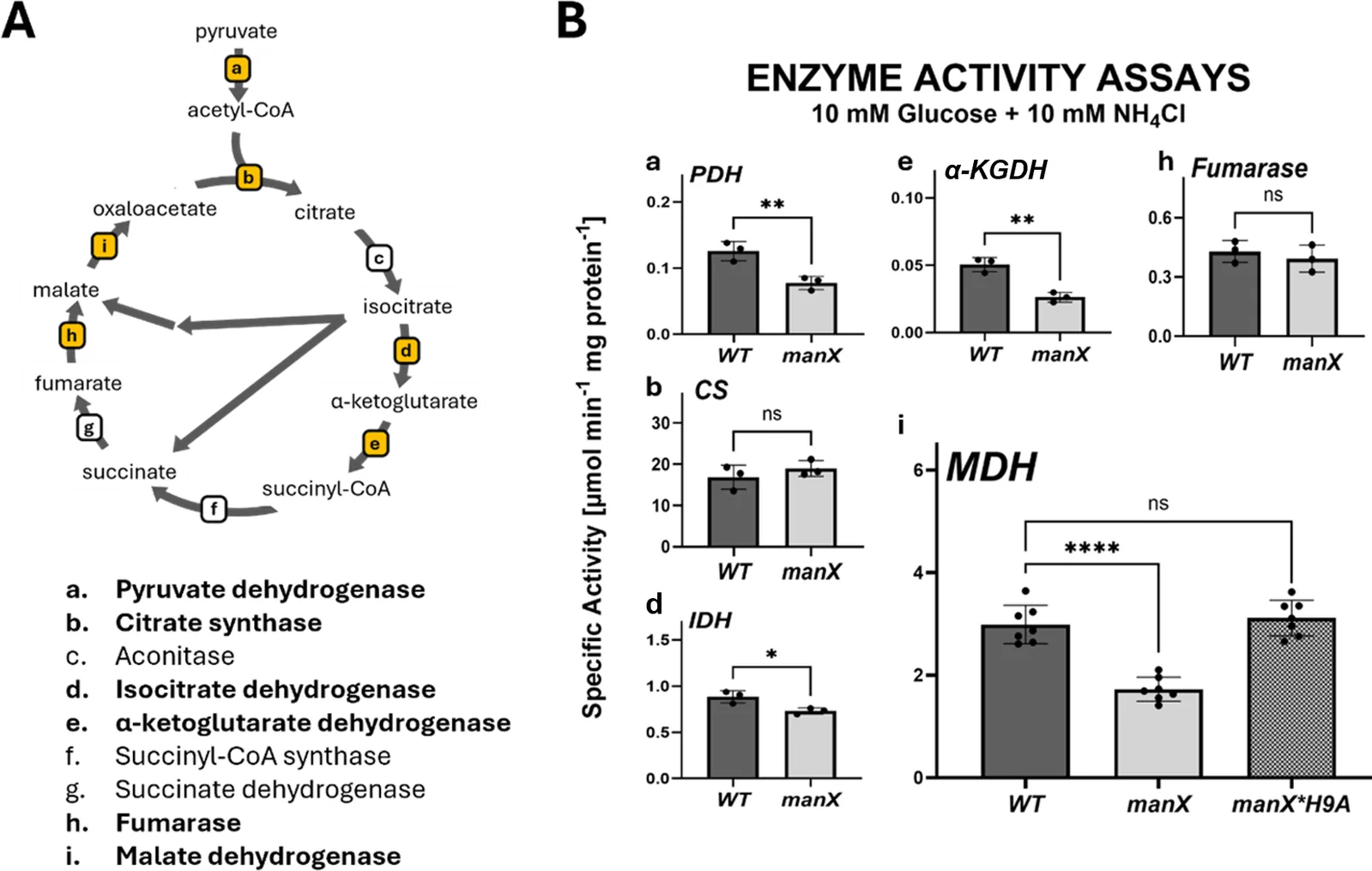
Activity of TCA cycle enzymes. (**A**) Diagram of the TCA cycle with the assayed enzymes being highlighted in yellow boxes as follows: (a) pyruvate dehydrogenase (PDH), (b) citrate synthase (CS), (d) isocitrate dehydrogenase (IDH), (e) α-ketoglutarate dehydrogenase (α-KGDH), (h) fumarase, and (i) malate dehydrogenase (MDH). (**B**) Enzyme activity assays of TCA cycle enzymes were performed using cell lysates of wild-type, *manX* mutant, and manX*H9A strain. Assays were normalized to total protein content. Data are averages (±SEM) from at least three independent cultures. Statistical significances were assessed by one-way ANOVA (for MDH) or Student’s *t*-test (other enzymes), with asterisks indicating the following level of significance: ****P ≤ 0.0001, **P ≤ 0.01, *P ≤ 0.05, and ns, not significant.

Our previous work had shown that *R. leguminosarum* PTS^Ntr^ mutants exhibited reduced growth, with mean generation times relevant for this study summarized in Table S1 and representative growth curves plotted in Fig. S1 (17). In particular, the Rlv3841 *manX* mutant displayed as well reduced activities of malate dehydrogenase (MDH) and α-ketoglutarate dehydrogenase (α-KGDH), indicating that ManX is a key factor required for full activation of the TCA cycle (17). To further define the role of ManX in TCA cycle function, in this study, we measured the activities of additional TCA cycle enzymes (Fig. 2A). In addition to confirming reduced activity of MDH and α-KGDH, we tested additional enzymes: fumarase hydratase, citrate synthase (CS), isocitrate dehydrogenase (IDH), and pyruvate dehydrogenase (PDH), which catalyzes the intermediate reaction linking glycolysis to the TCA cycle (Fig. 2B). Enzyme activity assays were performed on crude extracts of Rlv3841 wild-type and *manX* mutant strains. The reaction kinetics were assessed by monitoring substrate consumption over time. All bacterial strains were grown in universal minimal salts (UMS) medium supplemented with 10 mM glucose and 10 mM NH_4_Cl as nitrogen (N)-excess conditions.

The specific activities of CS and fumarase (Fig. 2B, panels b and h) were not affected in the *manX* mutant. However, the activity of PDH decreased by ~40%, IDH by ~17%, α-KGDH by ~54%, and MDH by ~40% (Fig. 2B, panels a, d, e, and i, respectively). The manX*H9A mutant (strain OPS1012), which mimics the non-phosphorylated state of ManX (His9 replaced by an alanine residue), had previously shown wild-type growth across diverse carbon sources and wild-type rates of oxygen (O_2_) consumption (17). We therefore assayed MDH activity and observed no change in the manX*H9A mutant relative to the wild-type (Fig. 2B, panel i). Collectively, our results indicate that ManX modulates the dehydrogenase enzymes of the TCA cycle, and its non-phosphorylated state is sufficient for activation of the TCA cycle’s dehydrogenases. In the absence of ManX regulation, TCA dehydrogenase enzymes exhibit reduced activity.

However, our attempts to biochemically validate these interactions by expressing ManX protein from Rlv3841 or by using bacterial two-hybrid assays (BACTH) in *Escherichia coli* were inconclusive. ManX aggregated and precipitated during purification and size-exclusion chromatography under all tested purification conditions. In addition, BACTH assays did not detect a direct protein interaction between Rlv3841 ManX and MDH or between ManX and SucA (Fig. S2). Although negative BACTH results can have several explanations (e.g., fusion geometry, absence of required cofactors, or expression level), the strong tendency of ManX to aggregate suggests that misfolding or aggregation of the ManX fusion protein in *E. coli* may limit the detection of this direct protein binding.

### The *hprK* mutant shows reduced oxidative metabolism

**Fig 3:**
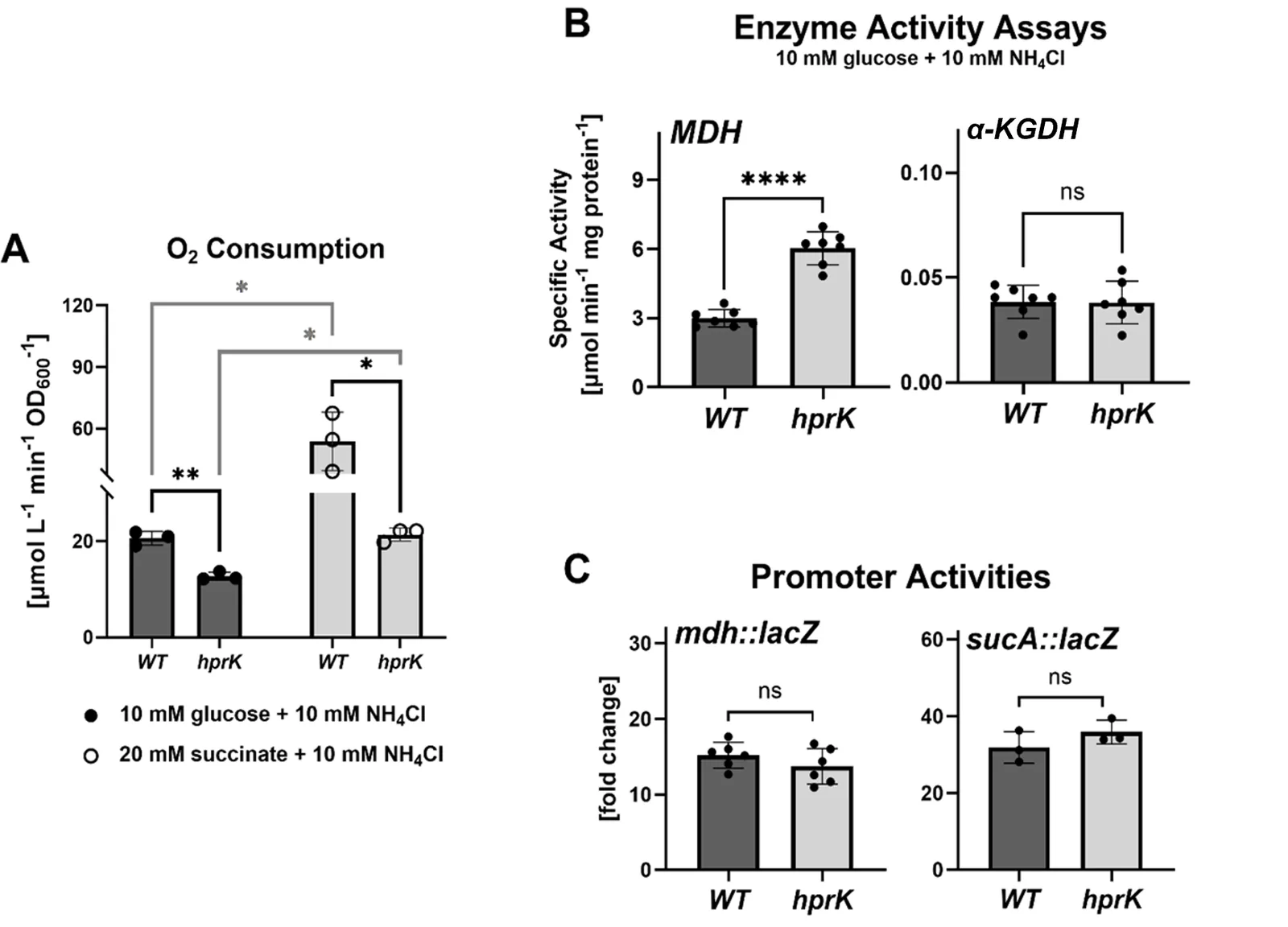
Metabolic phenotypes associated with the loss of HprK. (**A**) Central metabolic activity of the *hprK* mutant assessed indirectly by O_2_ consumption rates. The strains were grown in liquid UMS cultures supplemented with 10 mM NH_4_Cl as nitrogen source and either 10 mM glucose (dark gray bars) or 20 mM succinate as carbon source (light gray bars). (**B**) Enzyme activity assays of MDH and α-KGDH were performed in cell lysates of wild-type and *hprK* mutant grown in UMS cultures with 10 mM glucose and 10 mM NH_4_Cl and normalized to total protein contents. (**C**) Promoter activities for the genes encoding *mdh* and *sucA* were measured using the *lacZ* reporter assay on cultures grown on UMS with 10 mM glucose and 10 mM NH_4_Cl. Values are shown as the fold change relative to the strain-specific background rate of the promoter-less *lacZ* plasmid. Data are averages (±SEM) from at least three independent cultures. Statistical significances were assessed by Student’s *t*-test, with asterisks indicating the following level of significance: ****P ≤ 0.0001, **P ≤ 0.01, *P ≤ 0.05, and ns, not significant. In panel A, black asterisks are comparisons between strains grown in the same conditions, whereas gray asterisks indicate comparisons of the same strain grown under different carbon sources, done as a separate *t*-test.

As an aerobe, *R. leguminosarum* oxidizes TCA cycle intermediates to facilitate growth (31). Therefore, O_2_ consumption can serve as a proxy of aerobic respiratory activity and oxidative metabolism in free-living cell cultures. The Rlv3841 *hprK* mutant exhibited lower growth rates on various carbon sources (17), and we now show that this growth defect is accompanied by a reduced O_2_ consumption rate when it is grown under N-excess conditions (10 mM NH_4_Cl, supplemented with either 10 mM glucose or 20 mM succinate as carbon sources) (Fig. 3A). As seen in the wild-type, the O_2_ consumption rate for the *hprK* mutant is higher when succinate is used as the sole carbon source (Fig. 3). This can be explained by the fact that, unlike glucose, which is catabolized via the Entner-Doudoroff (ED) pathway, succinate feeds directly into the TCA cycle. Other studies had also confirmed that supplying succinate or other dicarboxylates leads to increased O_2_ demand and a highly reduced redox state (5, 32). As expected under N-limiting conditions (0.5 mM NH_4_Cl), the overall O_2_ consumption rates were low both in the wild-type and *hprK* mutant (Fig. S3).

### MDH activity is increased in the *hprK* mutant

To determine whether the reduced O_2_ consumption rate observed in the *hprK* mutant could be associated with downregulated TCA cycle activity, we measured *in vitro* the enzyme activity of MDH and α-KGDH in this mutant background (Fig. 3B). Paradoxically, despite the overall slowdown of the TCA cycle, MDH activity was significantly upregulated in the *hprK* mutant, while α-KGDH activity showed the same rate as that of wild-type. Our results indicate that the HprK-mediated regulation on the TCA cycle appears to occur at the post-transcriptional level. This is supported by the absence of significant changes in the promoter activity of *mdh* and *sucA* (encoding the Enzyme 1 component of α-KGDH) in the *hprK* mutant (Fig. 3C). Promoter activities of both genes remain comparable to those observed in the wild-type, indicating that PTS^Ntr^ does not modulate their transcription. Instead, HprK is likely to influence carbon metabolism through post-transcriptional mechanisms, potentially by altering PTS^Ntr^ activity and the consequent intracellular accumulation of phosphorylated ManX, or via other direct regulatory effects on central carbon metabolic processes.

### PTS^Ntr^ regulation modulates extracellular and intracellular polymer content

**Fig 4:**
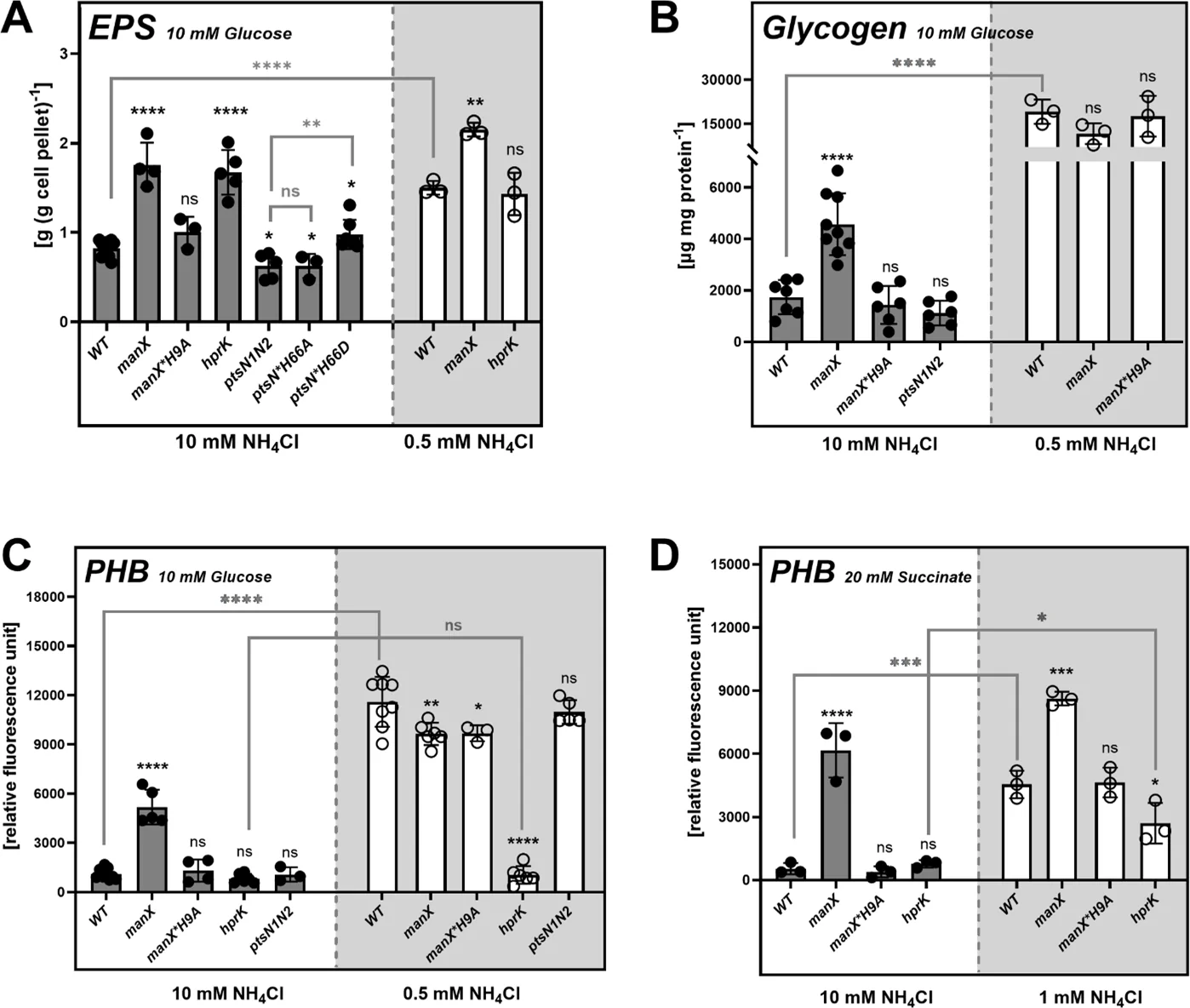
Carbon storage polymer accumulation in PTS^Ntr^ mutants under differing nitrogen availability and carbon sources. Quantification of three main carbon storage polymers in *R. leguminosarum*: (**A**) exopolysaccharide (EPS), (**B**) glycogen, and (**C** and **D**) polyhydroxybutyrate (PHB). The strains were grown in liquid UMS cultures supplemented with either 10 mM glucose (**A–C**) or 20 mM succinate (**D**), and with 10 mM NH_4_Cl (dark gray bars) or 0.5 mM NH_4_Cl (white bars). Data are averages (±SEM) from at least three independent cultures. Statistical significances were assessed by two independent one-way ANOVAs (for N-rich condition and N-limiting condition separately), with asterisks indicating the following level of significance: ****P ≤ 0.0001, ***P ≤ 0.001, **P ≤ 0.01, *P ≤ 0.05, and ns, not significant. Black asterisks are comparisons between strains grown in the same conditions, whereas gray asterisks indicate Student’s *t*-test comparisons between the specific strains being compared, as indicated by the connecting lines.

As PTS^Ntr^ influences the activity of TCA cycle enzymes, the disruption of PTS^Ntr^ regulation could lead to either overaccumulation or depletion of carbon storage polymers, depending on nutrient availability. Indeed, previous evidence indicated that PTS^Ntr^ regulates carbon storage in various gram-negative bacterial species (17, 20–22, 28, 29). Given the role of ManX in sustaining the activity of TCA cycle dehydrogenases, key PTS^Ntr^ regulatory components, particularly ManX, PtsN, or HprK, may together function as a control node linking central carbon metabolism with carbon storage. To investigate this possibility, we performed chemical assays to quantify and compare the accumulation patterns of carbon polymers in wild-type and PTS^Ntr^ mutant strains. EPS and two major intracellular carbon storage polymers in rhizobia, PHB and glycogen (Fig. 4A through C), were measured on cultures grown in UMS minimal medium supplemented with 10 mM glucose under two different nitrogen regimes: N-excess (10 mM NH_4_Cl) and N-limiting (0.5 mM NH_4_Cl). Additionally, the PHB content was also quantified in cultures grown in UMS with 10 mM succinate as a carbon source, as we reasoned that PHB storage might differ when the cells are grown on sugars versus an organic acid such as succinate, which feeds directly into the TCA cycle and can provide precursors for PHB biosynthesis (Fig. 4D).

Our results show that when carbon (glucose) was in surplus due to N-limitation (0.5 mM NH_4_Cl), Rlv3841 wild-type cells reallocated carbon into both intracellular (PHB and glycogen) and extracellular (EPS) storage polymers (Fig. 4A through C). This trend was also observed in all the mutants tested, consistent with a global metabolic response to nitrogen starvation. However, under N-excess conditions, unlike the wild-type, the *manX* mutant accumulated significant amounts of PHB, glycogen and EPS (Fig. 4A through C). This altered accumulation was not observed in the manX*H9A mutant (Fig. 4A through C), further supporting the sufficiency of the non-phosphorylated form of ManX for maintaining functional central metabolism.

To differentiate between effects arising from broad disruption of central carbon metabolism, as already seen in the *manX* mutant, versus effects caused by the loss of a single TCA cycle enzyme, we measured glycogen accumulation in the strain RU116. This strain carries a mutation in *sucD*, encoding the β-subunit of succinyl-CoA synthetase (32, 33). Under N-excess conditions, the RU116 mutant did not accumulate glycogen (Fig. S5). Upon N-starvation, glycogen increased in RU116 to a similar level as the wild-type. In this respect, the *sucD* mutant did not display the altered glycogen accumulation pattern seen in the *manX* mutant.

We next investigated whether the other output protein PtsN similarly contributes to the regulation of carbon storage. In particular, the phosphorylated form of this protein had previously been implicated in the regulation of EPS production (17). Intracellular polymer concentrations were not altered in the *ptsN1N2* mutant, with PHB (Fig. 4C) and glycogen (Fig. 4B) levels comparable to those of wild-type. Consistent with previous findings (17), the phenotype observed for *ptsN1N2* confirmed the reduction of EPS production (Fig. 4A). We also show that the ptsN*H66D mutant (strain OPS1104), a phosphomimetic version of PtsN (His66 replaced by a glutamate residue), enhanced EPS production beyond wild-type levels, whereas ptsN*H66A (strain OPS1102), a permanently non-phosphorylated PtsN, exhibited reduced EPS comparable to the original *ptsN1N2* mutant (Fig. 4A). The influence of PtsN in EPS biosynthesis was further supported by complementation assays of the *npr* mutant with NPr variants carrying amino acid substitutions at the two known phosphorylation sites His17 (PtsP-dependent) and Ser48 (targeted by HprK). The original *npr* mutant showed dry colony morphology when grown on agar plates, indicative of reduced EPS production (17). This same phenotype was observed when the *npr* mutant was now complemented with an NPr version carrying His17-to-alanine (H17A, non-phosphorylated) (Fig. S4B and F) or the NPr double mutation His17A and Ser48-to-alanine (S48A, non-phosphorylated) (Fig. S4D and H). In contrast, complementation with NPr carrying a S48A mutation, which prevents phosphorylation only by HprK, resulted in very wet colony morphology (Fig. S4C and G), in agreement with the *hprK* mutant EPS overproduction phenotype. Together, these results are consistent with HprK-mediated Ser48 phosphorylation of NPr inhibiting His17 phosphorylation (illustrated in Fig. 1A), and this is required for the induction of EPS biosynthesis.

Given the altered accumulation of carbon storage polymers observed in the *manX* mutant, we also assessed the *hprK* mutant. Under N-excess conditions, the *hprK* mutant accumulates EPS (Fig. 4A) but not PHB (Fig. 4C). Furthermore, unlike wild-type cells and other PTS^Ntr^ mutants tested, the *hprK* mutant failed to accumulate PHB under N-limiting conditions when glucose was the sole carbon source (Fig. 4C). Strikingly, the *hprK* mutant, which did not accumulate PHB when grown in glucose, built up PHB when grown in succinate under N-limiting conditions (Fig. 4D). This pattern differs from that of wild-type cells grown under N-limiting conditions, which accumulate less PHB when succinate is the carbon source than when glucose is, and from the *manX* mutant, which accumulates PHB regardless of the carbon source and nitrogen status.

### Raman spectroscopy reveals altered biomolecule accumulation in PTS^Ntr^ mutants

The results from chemical assays were further validated using single-cell Raman spectroscopy (SCRS), a non-invasive technique to monitor intracellular metabolites in their native cellular context based on their vibrational fingerprints (34). We quantified the PHB-associated peak at 1,735 cm^−1^ (35), glycogen-associated peaks at 850 cm^−1^, and 1,050 cm^−1^ (35), as well as spectral regions associated with pyrimidines (765–795 cm⁻¹) (36, 37) and amides (1,645–1,676 cm) (36–38). Pyrimidine-containing nucleotides are central components of DNA and RNA and play a fundamental role in cellular metabolism and biosynthesis, while amide bonds constitute the backbone of peptides and proteins, providing a direct proxy for cellular protein content and nitrogen allocation. The wild-type and PTS^Ntr^ mutants *manX* and *hprK* were grown on glucose (10 mM) as the carbon source and NH₄Cl (10 mM) as the nitrogen source. In addition, Rlv3841 wild-type strain was grown under N-limiting conditions in UMS containing 0.5 mM NH₄Cl to enable comparisons of macromolecular allocation strategies across contrasting nitrogen regimes.

**Fig 5:**
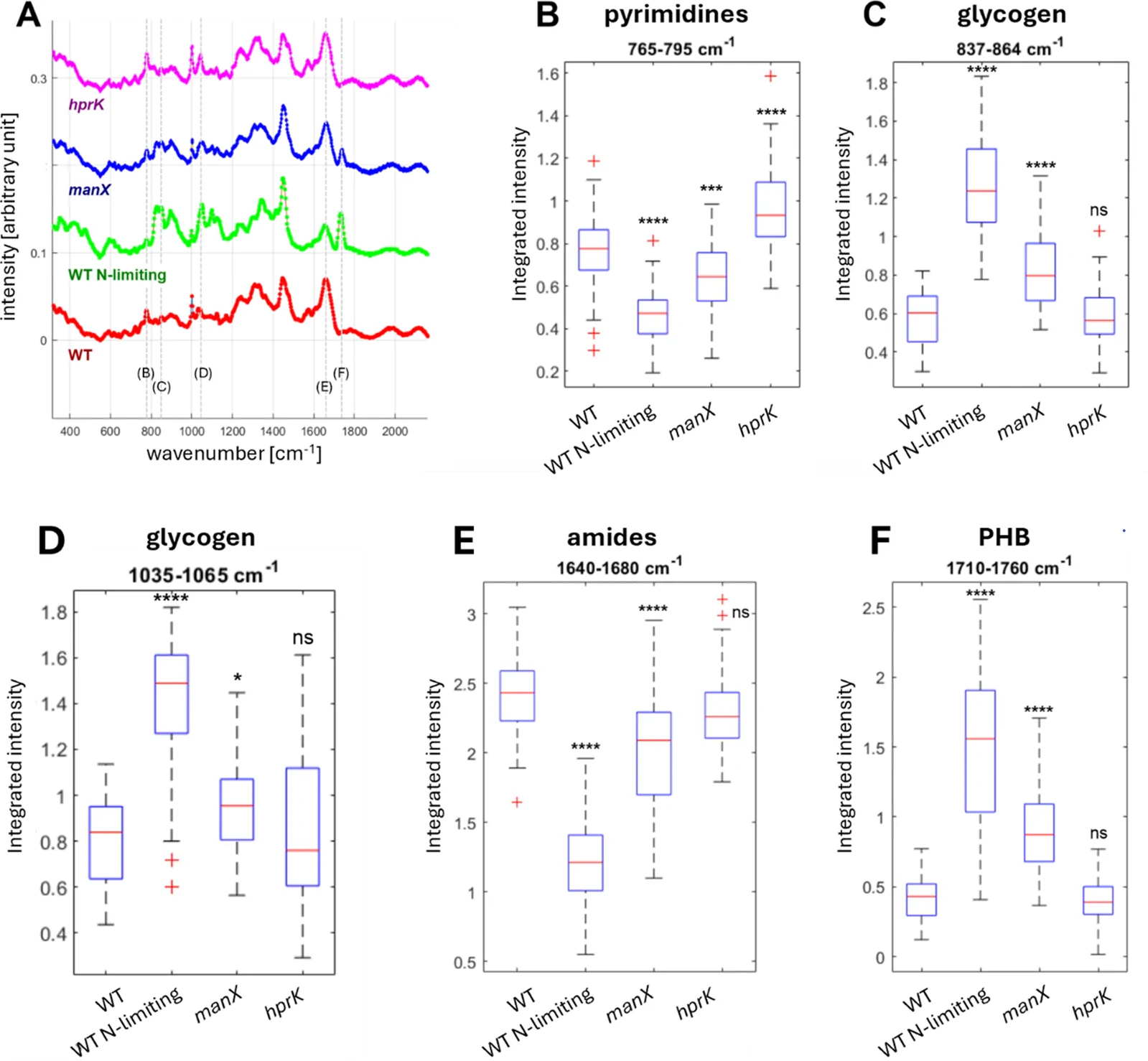
Quantification of biomolecular features using integrated band intensities of single-cell Raman spectra. (**A**) Averaged single-cell Raman spectra of wild-type (red), *manX* (blue), and *hprK* (magenta) grown under N-excess conditions (10 mM glucose + 10 mM NH_4_Cl) and wild-type under N-limiting conditions (green; 10 mM glucose + 0.5 mM NH_4_Cl). (**B–F**) Box plots of integrated areas under selected Raman bands for quantitative analysis. Integrated intensity of each band was calculated after baseline correction, with bands’ identity as such: 765–795 cm^−1^ (pyrimidine ring breathing), 837–864 cm^−1^ (glycogen), 1,035–1,065 cm^−1^ (glycogen), 1,640–1,680 cm^−1^ (amides), and 1,710–1,760 cm^−1^ (PHB). Data are averages (±SEM) from at least 60 replicates, and red crosses are outliers. Statistical significances were assessed by one-way ANOVA, with asterisks indicating the following level of significance: ****P ≤ 0.0001, ***P ≤ 0.001, *P ≤ 0.05, and ns, not significant.

As expected, wild-type cells grown under N-limiting conditions accumulated intracellular storage polymers (PHB and glycogen), consistent with an excess of carbon availability relative to metabolic demand (Fig. 5A through F). This metabolic imbalance was accompanied by reduced amides and pyrimidines, as nitrogen scarcity limits the synthesis of amino acids and nucleotides (39). Even when nitrogen was available, the *manX* mutant built up glycogen and PHB, while also reducing the levels of amides and pyrimidines, mimicking wild-type cells grown under N-limitation. In contrast to *manX*, the *hprK* mutant accumulated nucleotides but maintained low levels of PHB, glycogen, and amides comparable to those of wild-type under N-excess conditions (Fig. 5A through F).

### Differential expression of *pckR* and its target genes in the *hprK* mutant is consistent with gluconeogenic bias under glycolytic conditions

The combined phenotypes of growth defects on both glucose and succinate, elevated MDH activity, and altered accumulation of EPS and PHB in the *hprK* mutant suggested a shift away from the Entner-Doudoroff (ED)/glycolytic flux toward the TCA cycle/gluconeogenesis. Given that the LacI-type transcriptional regulator PckR (PEP carboxykinase regulator) has been predicted to repress ED genes and activate gluconeogenic genes in central metabolic control in rhizobia (40, 41), we measured the expression of *pckR* and selected PckR target genes by quantitative PCR (qPCR) during growth on glucose and succinate to test whether deregulated *pckR* expression could account for the *hprK* mutant phenotypes. These PckR target genes analyzed included on the gluconeogenic side *pckA* and *fbaB* (encoding respectively phosphoenolpyruvate carboxykinase [PEPCK] and fructose-1,6-bisphosphate aldolase [FBA]) and *zwf* and *pgl* (encoding glucose-6-phosphate dehydrogenase [G6PD] and 6-phosphogluconolactonase [6PGL]) on the ED side.

**Fig 6:**
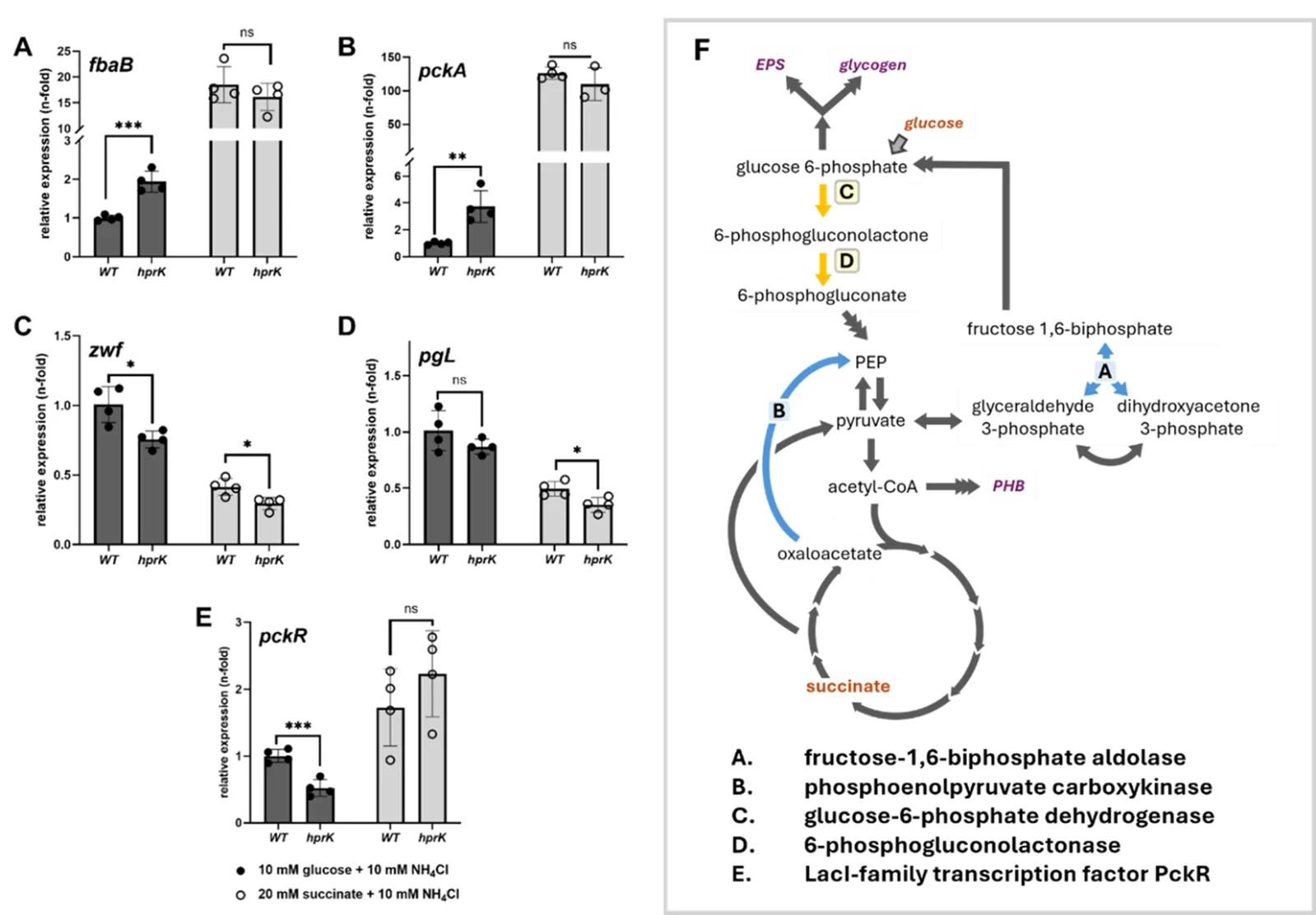
qPCR analysis of *pckR* and its target genes in the *hprK* mutant compared to wild-type. Relative transcript level of gluconeogenesis genes *fbaB* (**A**) and *pckA* (**B**), the ED pathway genes *zwf* (**C**) and *pgl* (**D**), and their transcriptional regulator *pckR* (**E**) are measured using quantitative PCR (qPCR). (**F**) Schematic representation of central carbon metabolism indicating the position of the analyzed enzymes within gluconeogenesis (A and B; blue) and glycolysis (C and D; yellow). Carbon storage compounds measured in this work are shown in purple, and carbon sources are marked in orange. Only selected intermediates are shown. The strains were grown in liquid UMS cultures supplemented with 10 mM NH_4_Cl as the nitrogen source and either 10 mM glucose (dark gray bars) or 20 mM succinate (light gray bars) as the carbon source. Data are averages (±SEM) from at least three independent cultures. Statistical significances were assessed by Student’s *t*-test, with asterisks indicating the following level of significance: ***P ≤ 0.001, **P ≤ 0.01, *P ≤ 0.05, and ns, not significant.

When grown on glucose, the *hprK* mutant showed increased expression of gluconeogenic genes, with *pckA* and *fbaB* expressed at approximately 3.7-fold and 2-fold higher levels, respectively, than in the wild-type (Fig. 6A and B). In contrast, expression of the ED pathway gene *zwf* was slightly reduced, while no significant difference was observed for *pgl* (Fig. 6C and D). During growth on succinate, the expression levels of *pckA* and *fbaB* in the *hprK* mutant were comparable to those of wild-type. Interestingly, both genes were highly induced on succinate (approximately 19- and 120-fold, respectively), compared with glucose-grown cells. Regarding *zwf* and *pgL*, both genes showed a slight decrease in expression in the *hprK* background and were generally expressed at lower levels in succinate-grown cultures (Fig. 6C and D). The transcript levels of *pckR* were approximately twofold lower in the *hprK* mutant grown on glucose and were similar to that of the wild-type grown on succinate (Fig. 6E).

## DISCUSSION

The regulation of carbon and nitrogen metabolism in symbiotic rhizobia is critically important for the success and efficiency of BNF (17). In Alphaproteobacteria, PTS^Ntr^ regulation ensures a balanced central metabolic activity and supply of energy based on nitrogen availability. Rhizobia are known to accumulate carbon polymers as energy reserves and as a mechanism to maintain redox balance during nitrogen starvation (42, 43). In line with this, we observed accumulation of two key internal polymers, PHB and glycogen, under such conditions in free-living *R. leguminosarum* cells. EPS production, which shares sugar biosynthetic precursors with glycogen, was also high. A decreased level of pyrimidines was also detected, and this is consistent with data from *E. coli* and *Saccharomyces cerevisiae*, where growth under nitrogen or carbon limitation similarly leads to diminished nucleotide abundance (39).

**Fig 7:**
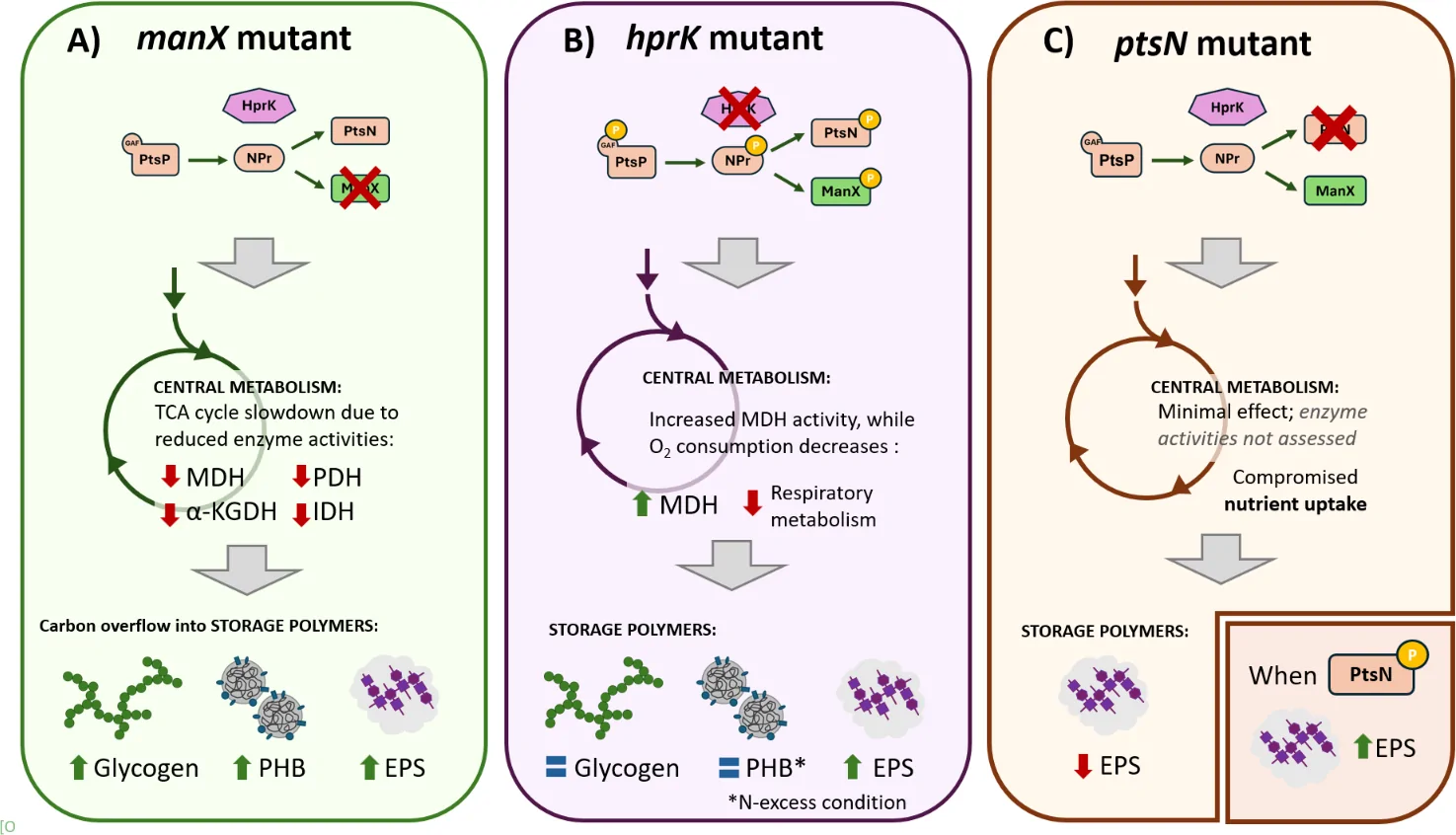
Proposed working model summarizing the effects of (**A**) *manX*, (**B**) *hprK*, and (**C**) *ptsN* disruption on carbon metabolism and storage polymers. (**A**) Loss of *manX* is linked to decreased activities of multiple TCA cycle dehydrogenases (including the measured rates of MDH, α-KGDH, PDH, and IDH). This reduced TCA cycle capacity may decrease carbon flow throughout the TCA cycle and favor the diversion of excess carbon and reductants into storage polymers, as measured by EPS, PHB, and glycogen. (**B**) The mutation of *hprK* is expected to promote phosphorylation of the downstream regulators PtsN and ManX, although the mechanistic details are not yet resolved in this study. Consistent with phosphorylated PtsN inducing EPS production, the *hprK* mutant accumulates EPS. The *hprK* mutant also shows increased MDH activity, elevated pyrimidine levels (not shown in the figure), and increased expression of gluconeogenic genes, which together support a bias toward the gluconeogenic pathway in this mutant. (**C**) The *ptsN* mutant, previously shown to be compromised in nutrient uptake while exhibiting minimal alterations in central metabolism, accumulates less EPS than the wild-type. Phosphorylated PtsN enhances EPS biosynthesis.

In *R. leguminosarum*, ManX and PtsN integrate environmental cues, particularly nitrogen and carbon availability, to control downstream metabolic outputs (17, 29). In this study, we demonstrate that the absence of ManX leads to reduced activity of TCA cycle dehydrogenases, followed by the accumulation of key intracellular carbon-storage polymers (glycogen and PHB) and the extracellular carbon polymer EPS. The *manX* mutant also exhibited reduced levels of amides and pyrimidines, which resemble N-starved wild-type cells (18). Our findings are summarized in Fig. 7. The phenotype observed in the *manX* mutant when grown on sugar is likely caused by overflow carbon metabolism. With the TCA cycle flux reduced, while sugar intake remains relatively high, excess carbon is diverted into biosynthetic pathways for carbon (glycogen and EPS) and energy (PHB) reserve polymers. Indeed, the accumulation of PHB and lipids has been reported across diverse bacterial species as a mechanism to buffer excess energy and reductants, which typically occurs under nutrient imbalance (44–47), as observed in the nitrogen-starved wild-type strain.

The reduction in amides and pyrimidines observed in the *manX* mutant can be attributed to similar underlying regulatory mechanisms. The overall slowdown of the TCA cycle likely reduces the availability of intermediates such as α-ketoglutarate, which is required for glutamate synthesis and ammonia assimilation. The limited fumarate or oxaloacetate availability contributes to less aspartate, which serves as a carbon backbone in *de novo* pyrimidine synthesis. Overall, these results support previous hypothesis (17, 29) that ManX serves as the branch of this integrated PTS^Ntr^ that is involved in carbon regulation.

As a potential explanation for the reduced TCA cycle dehydrogenase activities, we assessed the expression patterns of genes encoding MDH and α-KGDH. However, the transcript levels of malate and α-ketoglutarate dehydrogenase components remain unchanged in the *manX* mutant (17), further excluding transcriptional regulation. A strain with a permanently non-phosphorylated version of ManX (ManX*H9A) restored growth, enzyme activities, and polymer accumulation to the wild-type level, suggesting that ManX is unlikely to exert its regulation through phosphorylation. Taken together, these findings suggest that ManX predominantly influences dehydrogenase activities at the post-transcriptional level. Prior evidence showed direct interaction between ManX and α-KGDH (E1 component SucA) in *B. melitensis* when assayed using Y2H (19) and EIIA^Ntr^ (PtsN homolog) with the E1 subunit of PDH in *P. putida* by co-immunoprecipitation (18). Therefore, ManX function is potentially mediated through protein-protein interactions.

The observed slowdown of the TCA cycle, accompanied by increased carbon allocation to storage compounds despite unaltered amino acid transport in the *manX* mutant (17), suggested that ManX affects overall TCA cycle activity and that the phenotypes observed in the *manX* mutant could not be explained by defects in one or a few TCA cycle enzymes. This hypothesis was reinforced by comparison with strain RU116, which carries a mutation in *sucD*, encoding the β-subunit of succinyl-CoA synthetase (32, 33). According to previous work, although RU116 exhibits reduced growth, low succinyl-CoA synthetase activity, and strong upregulation of MDH (33), it showed reduced amino acid uptake (33) and did not accumulate lipids (32). Our results here further show that the RU116 mutant did not accumulate glycogen when N was available either (Fig. S5). Therefore, when the TCA cycle is blocked due to direct enzyme impairment, cells limit carbon and reductant buildups by reducing cycle flux and redirecting the TCA intermediates. In contrast, disruption of *manX* leads to a global imbalance in carbon metabolism, resulting in carbon overflow into storage compounds.

In previous work, we showed that PtsN acts on EPS biosynthesis through its interaction with the ChvG/I two-component system (17). We now show that the phosphorylated state of PtsN is essential for this function, as evidenced by differences in EPS accumulation between the non-phosphorylatable (H66A) and phosphomimetic (H66D) PtsN variants. While the ptsN*H66A mutant shows EPS levels comparable to that of the original *ptsN* mutant, the ptsN*H66D strain accumulates EPS in slightly higher amounts than Rlv3841 wild-type. The other polymers tested were not altered in the *ptsN* mutant, suggesting that the role of PtsN in carbon metabolism is exclusively linked to the control of EPS biosynthesis or secretion. In the *hprK* mutant, the preferential accumulation of carbon into EPS, but not glycogen, is likely driven by the activity of PtsN~P, which is the predominant state of PtsN in this mutant (19, 48), leading to the upregulation of EPS production. This was further supported by EPS surface phenotypic assays on agar plates using NPr variants containing substitutions at the Ser48 and His17 phosphorylation sites, showing that the histidine phosphorylation, leading to PtsN~P, is the dominant NPr phosphorylation site (Fig. S4).

Like the regulation exerted by non-phosphorylated ManX, the regulatory role of HprK cannot be easily explained by a single target. The *hprK* mutant shows a severe growth defect on various carbon sources, including glucose or succinate (Table S1; Fig. S1) (17, 22), along with reduced O_2_ consumption (17). The *hprK* mutant also shows EPS overproduction, elevated nucleotide levels, and increased MDH activity. Under N-limitation, the *hprK* mutant failed to accumulate PHB when the carbon source is glucose, but it did when grown on succinate. This effect might be due to the *hprK* mutant biasing carbon flow toward gluconeogenesis and TCA cycle activity under conditions that normally favor ED metabolism. MDH links the TCA cycle to gluconeogenesis by catalyzing the conversion of malate to oxaloacetate (Fig. 1B). Increased MDH activity could therefore elevate oxaloacetate levels, leading to enhanced aspartate formation and subsequent nucleotide biosynthesis, which may account for the elevated pyrimidine levels observed in this mutant. In addition, oxaloacetate may be converted to PEP via PEPCK, directing carbon flow through gluconeogenesis toward glucose-6-phosphate, a central metabolic intermediate that supplies precursors for both glycogen and EPS biosynthesis.

The relative shift toward the gluconeogenesis pathway in the *hprK* mutant is also supported by its PHB accumulation pattern. In rhizobia, glucose is normally processed primarily through the ED/PPP (pentose phosphate) pathways, and PHB synthesis requires a route from the carbon source to acetyl-CoA and a sufficient NADPH supply during nitrogen limitation (5, 49). Therefore, the lack of PHB accumulation by the *hprK* mutant during growth on glucose under nitrogen limitation, despite PHB accumulation on succinate, suggests a defect in ED/PPP-dependent PHB synthesis from glucose. It also indicates a potential carbon shift toward a more gluconeogenic state, rather than the typical ED-dominated metabolic state observed during growth on glucose.

In rhizobia, a well-characterized regulator that coordinates the balance between ED/glycolytic metabolism and TCA cycle/gluconeogenesis is PckR, a LacI-family transcriptional regulator that represses the ED pathway (*zwf–pgl–edd*, *eda2*, *mgsA*) and activates the gluconeogenic genes *pckA* and *fbaB* upon DNA binding (40, 41). We therefore measured transcript levels of *pckR* and its target to assess whether the misregulated central carbon control in the *hprK* mutant arises from altered PckR regulation. Our results suggest that the mutant shifts central metabolism toward gluconeogenesis under typically glycolytic conditions (i.e., glucose growth), while the transcriptional program associated with succinate utilization is largely intact. Altogether, these data are consistent with PckR being a plausible participant in the misregulated central carbon metabolic network caused by the *hprK* mutation. However, since *pckR* transcript abundance changes only modestly, and only when cultures are grown on glucose but not on succinate, these results do not demonstrate whether HprK directly regulates *pckR*. Instead, HprK likely influences how the PckR regulatory network responds to different carbon sources, either indirectly by altering the cell’s metabolic state or directly by modulating *pckR* expression under certain conditions.

Loss of HprK was expected to relieve serine-dependent inhibition of NPr histidine phosphorylation and thus favor phosphorylation of both downstream effectors ManX and PtsN. The increased EPS production in the *hprK* background, along with the *npr* complementation data, is consistent with enhanced His-phosphorylation through the PtsN branch. However, the *hprK* mutant does not phenocopy the effects of ManX perturbation. Although our study indicates that ManX is functionally active in its non-phosphorylated form, the growth defect of the *hprK* mutant cannot be fully explained by a simple reduction in the unphosphorylated ManX pool, as the *hprK* dehydrogenase readouts do not match the *manX* mutant phenotype. This *hprK* phenotype might also reflect the additional metabolic burden resulting from hyperphosphorylated PtsN, as this state could lead to an overactivation of ABC transport and misregulation of ion homeostasis, among other effects, that may contribute to the *hprK* growth defect. Thus, the *hprK* mutation results in an overall regulatory imbalance between PtsN and ManX output proteins, which in turn produces complex downstream effects that are more consistent with differential regulation than with a uniform increase in phosphorylation of both ManX and PtsN. Future studies will be needed to assign *hprK* mutant phenotypes to specific downstream PTS^Ntr^ EIIA components and to determine whether HprK has additional regulatory functions beyond those mediated by ManX or PtsN.

### Conclusion

Our findings demonstrate that PTS^Ntr^ coordinates carbon metabolism and storage through interconnected metabolic regulatory mechanisms. Central to this role is ManX, whose activity maintains the functionality of TCA cycle dehydrogenase enzymes. Loss of ManX disrupts the carbon flow throughout the TCA cycle, leading to the accumulation of all tested carbon storage polymers (glycogen, PHB, and EPS) and phenocopying nitrogen starvation even when nitrogen is available. While the regulatory effect on intracellular polymers appears to be mediated by ManX, analysis of PtsN variants supports a role for phosphorylated PtsN in promoting EPS production. This regulatory balance is controlled by NPr phosphorylation and HprK, which modulates the phosphorylation state of the PTS^Ntr^ output proteins. The metabolic phenotypes of the *hprK* mutant suggest a bias toward a gluconeogenic state during growth on sugars, confirming complex regulatory effects driven by increased phosphorylation of ManX and PtsN. Collectively, our results reveal a tightly integrated regulatory network linking central metabolism with carbon storage. Imbalanced PTS^Ntr^ regulation disrupts this coordination, slowing TCA cycle activity and driving atypical patterns of carbon polymer accumulation. Overall, these findings reinforce the role of PTS^Ntr^ as a central regulatory network controlling bacterial metabolic balance.

## MATERIALS AND METHODS

### Bacterial strains, plasmids, and culture conditions

This work was conducted using *R*. *leguminosarum* bv. viciae 3841 (Rlv3841) or derivative mutants listed in Table S2. *R. leguminosarum* was grown on tryptone yeast nutrient-rich medium or the minimal medium Universal Minimal Salts (UMS) supplemented with 10 mM glucose and 10 mM NH_4_Cl, unless otherwise stated (50, 51). *R. leguminosarum* was grown at 28°C supplemented with antibiotics at the following concentrations (μg mL^−1^): spectinomycin 100, streptomycin 500, and tetracycline 1 (liquid cultures) or 5 (agar plates). *E. coli* was grown on nutrient-rich Luria-Bertani medium at 37°C unless otherwise stated (52). *E. coli* cultures were supplemented with antibiotics at concentrations (μg mL^−1^) of ampicillin 100, kanamycin 20, spectinomycin 50, and streptomycin 25.

### TCA cycle enzyme assays

*R. leguminosarum* strains were grown in 50 mL UMS supplemented with 10 mM glucose and 10 mM NH_4_Cl to mid-exponential growth at an OD_600_ of 0.4–0.6. Bacterial cells were then washed and resuspended in 20 mM phosphate buffer, pH 7.2, containing 1 mM dithiothreitol, and cytosolic fractions were obtained by breaking the cells with FastPrep-24 5G Ribolyzer (MP Biomedicals), followed by centrifugation at 13,000 rpm to remove membranes and debris. Enzyme activities were assayed with a spectrophotometer at 28°C by monitoring the rate of change in absorbance per second upon the addition of the cell lysate and respective substrates. The enzyme rate was standardized by measuring the protein concentration of whole cells using Qubit Protein Assay Kits following the manufacturer’s instructions. The final compositions of the reaction mix and wavelength used for each enzyme assay were as follows: citrate synthase (200 mM Tris/HCl pH 8.1, 0.2 mM DTNB, 0.1 mM Acetyl-CoA, 0.5 mM oxaloacetate, measured at 412 nm) (53), isocitrate dehydrogenase (20 mM Tris/HCl pH 7.4, 3.3 mM MgCl_2_, 0.5 mM NADP, 0.5 mM DL-isocitric acid, measured at 340 nm) (54), α-ketoglutarate dehydrogenase (100 mM Tris/HCl pH 8.4, 0.1 mM CoA, 7.8 mM cysteine, 25 mM α-ketoglutarate, 2 mM NAD, measured at 340 nm) (53), fumarase (50 mM phosphate/NaOH pH 8, 50 mM L-malate, measured at 240 nm) (55), malate dehydrogenase (100 mM phosphate/NaOH pH 7.5, 0.2 mM NADH, 0.5 mM oxaloacetic acid, measured at 340 nm) (56), and pyruvate dehydrogenase (50 mM pH phosphate/NaOH 7.5, 3.5 mM NAD, 0.12 mM CoASNa, 4 mM cysteine, 0.3 mM thiamine pyrophosphate, 1.5 mM MgCl_2_, 2.5 mM sodium pyruvate, measured at 340 nm) (56). All assays were performed from at least three independent biological replicates. Negative controls (lysates without substrate or cofactor) were included to account for background activity.

### O_2_ consumption assays

To measure oxygen (O_2_) consumption, 25 mL liquid cultures at exponential phase were added to a glass universal with an OxyDot oxygen sensor and a magnetic stirrer and quickly sealed. O_2_ consumption was measured in real time every 15 s using a non-invasive O_2_ electrode from the OxySense 325I System (Industrial Physics) and analyzed with the OxySense GenIII program as previously described (17).

### Promoter activity assays

The promoter activity of *mdh* or *sucA* was measured using the β-galactosidase method (57, 58). To do this, cosmids containing the *lacZ* (β-galactosidase) reporter gene under the control of the *mdh* or *sucA* promoter (pRU3037 and pRU3068, respectively) were conjugated into *R. leguminosarum* (17) using the pRK2013 helper plasmid. A promoter-less plasmid containing only *lacZ* was included as an experimental control and baseline measure. Cultures were harvested during the exponential phase. Transcriptional activity was converted into Miller units (58). Activity is reported as fold-change relative to strain-specific background rates to adjust for the overall lower transcriptional activity of slow-growing strains.

### Quantification of carbon polymers

Quantification of intracellular and extracellular carbon polymers was performed on cells harvested at the late exponential phase (OD_600_ ~0.8–1.0), when these polymers typically accumulate. The key mutants selected for the study were *manX* and *hprK* mutants, as these are two PTS^Ntr^ strains that are the primary focus of this work. Additional mutants such as *ptsN1N2* or *sucD* were included only for specific polymer-condition combinations where prior work implicated their role in those pathways (17, 34), rather than as an exhaustive panel across all strains and conditions.

Intracellular PHB content was determined by flow cytometry after staining cells with the fluorescent dye Nile Red (NR). The median fluorescence intensity, expressed in the arbitrary fluorescent unit, has been reported to correlate linearly well with the bacterial PHB content (59, 60). Briefly, *R. leguminosarum* strains were grown in 10 mL UMS with 10 mM glucose as a carbon source and 10 mM ammonia (N-rich) or 0.5 mM ammonia (N-poor) to an OD_600_ of 0.8–1. Bacterial cells were harvested and washed with phosphate-buffered saline (PBS) buffer and then permeabilized by treatment with 35% ethanol for 15 min. They were diluted in PBS to reach a concentration of 5 × 10^6^ cells, stained with 0.01% (wt/vol) NR stock solution (20 µL for 1 mL sample) for 30 min under dark conditions, and then analyzed on the flow cytometer (Amnis CellStream) with the excitation 488 nm wavelength of Argon Laser. Fluorescence was measured in channel F2, with a filter centered on a bandwidth of 585 nm. The data are analyzed with CellStream software. Strain LMB816 (*phaC1/phaC2* double mutant) was used as the negative control (32).

Glycogen was extracted from *R. leguminosarum* cultures following a modified version of the previously described protocol (61) by boiling it with 30% KOH for 1.5 h. Dissolved glycogen was then precipitated with 70% EtOH, spun down, and dried. The glycogen amount for each sample was normalized with the total protein content of the cells. For quantification, pelleted glycogen was resuspended in 10 mM acetate buffer, pH 4.75, and digested with amyloglucosidase (4 units/assay) and amylase (8 units/assay) for 1.5 h at room temperature. Aliquots of the digested samples were then assayed by standard hexokinase/glucose-6-phosphate dehydrogenase (G6P-DH/HK) assay coupled with NADP^+^ reduction (32). The assay reaction mix contained 265 mM triethanolamine buffer pH 7.6, 3.3 mM MgCl_2_, 10 mM ATP, 10 mM NADP, 0.5 units of G6P-DH/HK, and the sample. The increase in absorbance at 340 nm was read with the Omega FLUOstar plate reader. Strain OPS2031 (*glgA* mutant), deficient in glycogen biosynthesis, was used as the negative control, and glycogen content was calculated by comparing the absorbance (340 nm) with that of the glycogen standards.

EPS was quantified following the previously described protocol (17) from *R. leguminosarum* cultures grown in 50 mL UMS with 10 mM glucose and 10 mM NH_4_Cl to reach an OD of 1. The strain LMB310 (*pssA* mutant unable to produce EPS) was used as the negative control (62).

### Raman spectroscopy and analysis

Single-cell Raman spectra (SCRS) were acquired using a confocal Raman micro-spectrometer (HR Evolution 800, Horiba, Japan) equipped with a continuous 532 nm laser set to 80 mW. The laser beam was attenuated with a 10% neutral-density filter and focused onto the sample through a 100× objective (NA 0.9; Olympus, Japan). Spectra were recorded with a 300 lines/mm⁻¹ grating, with the spectral center set at 2,000 cm⁻¹. The Raman acquisition time was 12 s per cell. For each sample group, at least 30 SCRS were randomly acquired.

Pre-processing of raw spectra comprised quality control to remove saturated high-intensity spectra, cosmic-ray spike removal, spectral trimming (retaining only the fingerprint region, 320–1,850 cm⁻¹), baseline fitting (third-order polynomial with 100 points), and baseline correction. The spectra were then normalized such that the sum of all integrated peak intensities was equal to one.

SCRS from all experimental groups were combined pairwise (wild-type N-rich vs wild-type N-limiting; wild-type vs *manX* mutant; wild-type vs *hprK* mutant), and principal component analysis (PCA) was performed on the fingerprint region for each pair. For each pairwise analysis, we examined combinations of the top 10 principal components (e.g., Dim 1 vs Dim 2 and Dim 3 vs Dim 6) to identify the projection that best separated the two groups (maximizing inter-group distance on the score plots). Using the optimal Dim pair thus identified, we computed the loadings across Raman shifts for that projection and ranked the shifts by loading value (from +1 to −1), yielding an ordered list according to each shift’s contribution to group discrimination. By cross-referencing our in-house Raman spectral database, we assigned the key biomolecular contributors underlying the separations observed for each pair of strains.

### RNA extraction and real-time qPCR analysis

Rlv3841 cells were harvested by centrifugation at 4°C, lysed using FastPrep-24 5G, and their cell debris was removed by another round of centrifugation. RNA was extracted using the Qiagen RNeasy extraction kit, and gDNA was depleted by treatment with Invitrogen Turbo DNAse according to the manufacturer’s recommendations. cDNAs were generated from 0.5 μg of purified RNA using an Invitrogen SuperScript IV reverse transcriptase kit as per the manufacturer’s recommendations. Quantitative PCR reactions were prepared in 384-well qPCR plates containing 5 μL of Applied Biosystems PowerUp SYBR Green Master Mix, 250 nM of each primer, and 5 ng of RNA template, for a total volume of 10 μL per reaction, run using the StepOnePlus Real-Time PCR System. Four biological replicates were extracted for each condition, each with two technical replicates. The primer pairs used in the real-time qRT-PCR are listed in Table S3 and were initially tested for amplification efficiency and target specificity by generating a standard curve of amplification with the *rpoD* housekeeping gene. Relative expression of each individual gene was calculated using the Pfaffl method.

### Statistical analysis

For Raman analyses, PCA, computation of PCA loading matrices, and score-plot visualization/analysis were carried out in R (version 4.4.2) using the base “stats” package together with “dplyr” and “ggplot2.” All the other statistical analyses were carried out using GraphPad Prism 10.5.0 (GraphPad Software, La Jolla, CA, USA). Student’s *t*-tests and analysis of variance (ANOVA) were used to measure the significance of results. Where multiple comparisons were necessary, the one-way ANOVA (Tukey-Kramer method) was used. We took a *P-*value less than 0.05 to be statistically significant. Biological replicates taken from single colonies were sampled with two to three technical replicates each.

## Data Availability

All data supporting the findings of this study are available within the article and its supplemental material.

## SUPPLEMENTAL MATERIAL

Supplemental material aem.01073-26-s0001.docx Tables S1 to S3, Fig. S1 to S5, and supplemental methods.

ASM does not own the copyrights to Supplemental Material that may be linked to, or accessed through, an article. The authors have granted ASM a non-exclusive, world-wide license to publish the Supplemental Material files. Please contact the corresponding author directly for reuse.
