# Supplementary material for: Regulation of central carbon metabolism and storage polymers by PTSNtr in Rhizobium leguminosarum
Source: Appl Environ Microbiol. 2026 Sep 30;92(10):e01073-26. doi: 10.1128/aem.01073-26 (PMC13689476; doi:10.1128/aem.01073-26)
Supplement: Supplementary file 1 [file aem.01073-26-s0001.docx]

Regulation of central carbon metabolism and storage polymers by PTS^Ntr^ in *Rhizobium leguminosarum*

**Supplementary Information**

Olivia Tjahjono, Jingkai Wang, Evan Turner, Jürgen Prell, Wei E. Huang, Philip S. Poole, Carmen Sánchez-Cañizares

**Table S1**. **Mean generation time for PTS^Ntr^ mutants**. Values were obtained from previous work (1), with those strains used in this study shown in bold. Strains were grown in Universal Minimal Salt (UMS) supplemented with ammonia (NH_4_Cl 10 mM) as the nitrogen source and 10 mM glucose or 20 mM succinate as the carbon source. Values are expressed in hours obtained from at least three biological replicates ± SEM. Mean generation times slower than the Rlv3841 wild-type are highlighted in red, with increasing intensity indicating the magnitude of the difference (light red ~1h slower; medium red ~2h slower; dark red >3h slower).

| **Description** | **Strain** | **Mean Generation Time (hours)** | |
| --- | --- | --- | --- |
|  |  | **Glucose 10 mM** | **Succinate 20 mM** |
| **wild-type** | **Rlv3841** | 3.42 ± 0.11 | 3.44 ± 0.28 |
| *ptsP* | ptsP107 | 4.44 ± 0.16 | 5.65 ± 0.32 |
| *npr* | AA031 | 4.43 ± 0.09 | 4.04 ± 0.43 |
| ***ptsN1, ptsN2*** | **AA047** | 3.33 ± 0.16 | 4.34 ± 0.35 |
| ***ptsN1**H66A, *ptsN2*** | **OPS1102** | 3.42 ± 0.09 | 3.51 ± 0.11 |
| ***ptsN1**H66D, *ptsN2*** | **OPS1104** | 4.48 ± 0.50 | 4.41 ± 0.08 |
| ***manX*** | **LMB692** | 6.13 ± 0.12 | 6.15 ± 0.21 |
| ***manX**H9A** | **OPS1012** | 3.39 ± 0.11 | 3.46 ± 0.10 |
| ***hprK*** | **AA081** | 7.64 ± 0.17 | 5.48 ± 0.24 |

**Table S2**. List of strains and plasmids

| **Strains** | **Description** | **Reference** |
| --- | --- | --- |
| ***Escherichia coli*** | |  |
| DH5a | *supE44*, *hsdR17*, *recA*, *thi-1*, *ΔlacU169*(f801acZΔM15) *endA1*, *gyrA96*, *relA1* | (2) |
| ***Rhizobium leguminosarum*** | | |
| Rlv3841 | *R. leguminosarum* bv. *viciae*; Str^R^ derivative of strain 300 | (3) |
| AA047 | Rlv3841 *ptsN2* in-frame deletion  in  *ptsN1*::ΩSpec background (*ptsN1N2* double mutant) | (1) |
| LMB310 | Rlv3841 *pssA*::Tn5 mutant | (4) |
| LMB692 | Rlv3841 *manX* (RL0033) in-frame deletion mutant | (1) |
| LMB816 | Rlv3841 *phaC1* (RL2098)/*phaC2*(pRL100105) double mutant | (5) |
| OPS1012 | Rlv3841 with non-phosphorylatable ManX made by a single amino acid substitution at H9A | (1) |
| OPS1102 | Rlv3841 *ptsN2* with non-phosphorylatable PtsN1 made by single amino acid substitution at H66A | (1) |
| OPS1104 | Rlv3841 *ptsN2* with PtsN1 phosphomimetic made by single amino acid substitution at H66D | (1) |
| RU1448 | Rlv3841 *glgA* in-frame deletion mutant | (6) |
| AA081 | Rlv3841 *hprK*::ΩSpec | (1) |
| AA031 | Rlv3841 npr::ΩSpec | (7) |
| PtsP107 | Rlv3841 Tn5::*ptsP* | (4) |
| **Plasmids** |  |  |
| pRK2013 | Self-transmissible helper plasmid, Km^R^ | (8, 9) |
| pK18mobsacB | Wide range suicide vector, oriT (mobilisable), *sacB*; Kan^R^ | (10) |
| pRU3070 | *mdh::lacZ* | (11) |
| pRU3068 | *sucA::lacZ* | (11) |
| pOPS0186 | *ptsN1* PCR-amplified from Rlv3841 genomic DNA using primers oxp516/517 and cloned into the BACTH vector pUT18C via BamHI/KpnI restriction sites | (1) |
| pOPS0174 | C-terminal region of *kdpD* PCR-amplified from Rlv 3841 using primers oxp520/521 and cloned into pKNT25 via BamHI/KpnI | (1) |
| pOPS0179 | *manX* PCR-amplified from Rlv3841 using primers oxp529/530 and cloned into pKNT25 via BamHI/KpnI | This work |
| pOPS0193 | *manX* PCR-amplified from Rlv3841 using primers oxp529/530 and cloned into pUT18C via BamHI/KpnI | This work |
| pOPS0289 | *mdh* PCR-amplified from Rlv3841 using primers oxp634/635 and cloned into pKNT25 via BamHI/KpnI | This work |
| pOPS0291 | *sucA* PCR-amplified from Rlv3841 using primers oxp632/633 and cloned into pKNT25via BamHI/KpnI | This work |
| pOPS0292 | *mdh* PCR-amplified from Rlv3841 using primers oxp634/635 and cloned into pUT18C via XbaI/KpnI | This work |
| pOPS0295 | *sucA* PCR-amplified from Rlv3841 using primers oxp632/633 and cloned into pUT18C via XbaI/KpnI | This work |
| pKNT25 | BACTH vector for fusions to the N-terminus of fragment T25, Kana^R^ | Euromedex |
| pUT18C | BACTH vector for fusions to the C-terminus of fragment T18, Amp^R^ | Euromedex |
| pOPS0741 | *mdh* (Rlv3841) cloned into pOPINF with an N-terminal 3×FLAG and His-tag by BD (In-Fusion) cloning | This work |
| pOPS0114 | *manX* (Rlv3841) cloned into pOPINF with an N-terminal 3×FLAG and His-tag by BD (In-Fusion) cloning | This work |
| pOPS1067 | *manX* His9Ala cloned into pOPINF with an N-terminal 3×FLAG and His-tag by BD (In-Fusion) cloning; | This work |
| pOPINF | Multi-host expression vector from the pOPIN vector suite for high-throughput cloning and expression, Amp^R^ | (12) |
| pRK415 | IncP stable broad-host-range cloning vector; Tet^R^ | (13) |

**Table S3.** List of primers

| **Primers** | **Description** | **Sequence** |  |
| --- | --- | --- | --- |
| oxp6980 | RT-qPCR primer for *rpoD* | GATGAAGTCGATCGGCAATCTG | |
| oxp6981 | RT-qPCR primer for *rpoD* | GCTTCGACCATTTCCTTCTTGG | |
| oxp7570 | RT-qPCR primer for *zwf* | TACTACGTCTCGGTCGATGC | |
| oxp7571 | RT-qPCR primer for *zwf* | GCTTGTGGTCGTGGATCTTC | |
| oxp7572 | RT-qPCR primer for *pgl* | CTTGCAACCGAGAAGACCAG |  |
| oxp7573 | RT-qPCR primer for *pgl* | TCCGCTTCCATGGTGATGAT |  |
| oxp7592 | RT-qPCR primer for *pckA* | GGTTCGACGACACCCTTTTC |  |
| oxp7593 | RT-qPCR primer for *pckA* | GACTTCATCCCGAACGCTTC |  |
| oxp7594 | RT-qPCR primer for *fbaB* | ATATCTCCGGCGTCATCCTC |  |
| oxp7595 | RT-qPCR primer for *fbaB* | CGACCTTGATGCCTGGAATG |  |
| oxp7596 | RT-qPCR primer for *pckR* | ATCGCTACCAGGGCTATGTC |  |
| oxp7597 | RT-qPCR primer for *pckR* | CAGCGAAAGGAAATGCACTG |  |
| oxp529 | Forward for PCR of *manX* for cloning into BATCH vectors with KpnI site | TTTGGTACCCGGCGCCCGATATCCGGCACTT |  |
| oxp530 | Reverse for PCR of *manX* for cloning into BATCH vectors with BamHI site | TTTGGATCCCATGATCGGACTTGTGCTTGT |  |
| oxp632 | Forward for PCR of *sucA* for cloning into BATCH vectors with XbaI site | TTTTCTAGACATGCGCCGCGGACAGGCGGC |  |
| oxp633 | Reverse for PCR of *sucA* for cloning into BATCH vectors with KpnI site | TTTGGTACCCGACCGCCCAATGCATCCTCGA |  |
| oxp634 | Forward PCR of *mdh* for cloning into BATCH vectors with BamHI site | TTTGGATCCCATGGCGCGTAACAAGATCGC |  |
| oxp635 | Reverse for PCR of *mdh* for cloning into BATCH vectors with KpnI siteoxp | TTTGGTACCGCCTTGAGGGCAGGCGCGATGT |  |
| oxp0193 | Forward PCR of *manX* and *manX* His9Ala with 3xflag tag for BD into pOPINF | *AAGTTCTGTTTCAGG*GCCCGATGGACTACAAGGACCACGACGGTGACTACAAGGACCACGACATCGACTACAAGGACGACGACGACAAGATCGGACTTGTGCTTGT |  |
| oxp0195 | Reverse PCR of *manX* and *manX* His9Ala for BD into pOPINF | *CTGGTCTAGAAAGCT*TCATTTTCCGCTGAGCACACGG |  |
| oxp0780 | Forward PCR of *mdh* with 3xflag tag for BD into pOPINF | *AAGTTCTGTTTCAGG*GCCCGATGGACTACAAGGACCACGACGGTGACTACAAGGACCACGACATCGACTACAAGGACGACGACGACAAGGCGCGTAACAAGATCGC |  |
| oxp0782 | Reverse PCR of *mdh* for BD into pOPINF | *CTGGTCTAGAAAGCT*CTACTTGAGGGCAGGCG |  |
| oxp0781 | Forward PCR of *mdh* without tag for BD into pOPINF | *AAGTTCTGTTTCAGGGGCCCGATGGCGCGTAACAAGATC* |  |
| pOPINFrev | Reverse primer for pOPINF mapping | CCAGCCACCACCTTCTGATA |  |

*Restriction sites in primer sequences are underlined, and overlapping sequences for BD cloning are marked in italics.

**Figure S1. Growth curves of Rlv3841 PTS^Ntr^ strains**. The wild-type and mutants tested were grown in UMS supplemented with 10 mM NH_4_Cl as nitrogen source and either 10 mM glucose (A) or 20 mM succinate (B) as carbon source. Strains shown are Rlv3841wild-type (black), *ptsP* (red), *npr* (yellow), *ptsN1N2* (grey), *ptsN1N2**H66A (green)*, ptsN1N2**H66D (white-filled dots)*, manX* (purple), *manX**H9A (pink) and *hprK* (blue). The same experimental dataset presented in Figure S1 was used to generate these growth curves (1).

**9**

**8**

**7**

**6**

**5**

**4**

**3**

**1**

**2**

**Figure S2. Bacterial two-hybrid assay (BACTH) to test potential interaction between Rlv3841 ManX and TCA cycle dehydrogenase enzymes MDH and α-KDH.** 1. Positive control (KdpD-T25/T18-PtsN1), 2. Negative control (empty vector pKNT25/pUT18C), 3. Mdh-T25/T18-ManX, 4. ManX-T25/T18-Mdh, 5. Mdh-T25/T18-ManX, 6. ManX-T25/T18-SucA, 7. SucA-T25/T18-ManX, 8. SucA-T25/T18-ManX, 9. ManX-T25/T18-Mdh. Apart from the positive control, the tested combinations did not yield detectable positive signals.

**Figure S3. O_2_ consumption rates under N-limiting conditions for the *hprK* mutant and Rlv3841 wild-type.** The strains were grown in liquid UMS cultures supplemented with 0.5 mM NH_4_Cl as nitrogen source and either 10 mM glucose (dark grey bars) or 20 mM succinate as carbon source (light grey bars). Data are averages (±SEM) from 3 replicates. Statistical significances were assessed by Student’s t-test, with asterisks indicating the following level of significance: ** *P* ≤ 0.01, and ns, not significant.

**Figure S4. Complementation assay of the *R. leguminosarum* *npr* mutant with different *npr* phosphorylation variants grown on TY agar plates.** Colony morphology and surface phenotypes are as follows: (A, E) AA031 original *npr* mutant complemented with the wildtype gene: *npr*::ΩSpec [pRK415:*npr*], mucoid; (B, F) AA031 *npr*::ΩSpec [pRK415:*npr**H17A], dry; (C, G) AA031 *npr::*ΩSpec [pRK415:*npr**S48A], very wet; (D, H) AA031 *npr*::ΩSpec [pRK415:*npr**H17A*S48A], dry. Panels (E-H) are magnified images of colonies grown on plates (A-D) to better appreciate colony morphology and surface mucoidity. The phenotype of the *npr* mutant complemented with [pRK415:*npr*] resembles that of wild-type (A, E), while complementation with [pRK415:*npr**H17A] (B, F) and [pRK415:*npr**H17A*S48A] (D, H) resulted in dry *npr* mutant-like colonies, as shown in previous work (1).

**Figure S5. Glycogen quantification in the strain RU116 (*sucD* mutant) and the wild-type.** The strains were grown in liquid UMS cultures supplemented with 10 mM glucose as a carbon source and either 10 mM NH_4_Cl (dark grey bars) or 0.5 mM NH_4_Cl (light grey bars). Data are averages (±SEM) from 3 replicates. Statistical significances were assessed by Student’s t-test, with asterisks indicating the following level of significance: ** *P* ≤ 0.01, and ns, not significant.

**SI Materials and Methods**

**Cloning details:** BACTH plasmids were constructed by amplifying the proteins tested with the corresponding primers listed in Table S3. The PCR products were cloned in frame into the BamHI/KpnI sites of pUT18C and pKNT25, generating the constructs listed in Table S2. These plasmids were confirmed by PCR sequencing with primers used in previous work, M13/pKNT25–258R and pUT18C-711F/pUT18C-804R (1). The complementing plasmids for the *npr* mutant were cloned following the procedures described in (6). For heterologous protein expression of Rlv3841 ManX and MDH in *E. coli*, template genomic DNA from the wildtype was used to amplify the *manX* (RL0033, 402 bp) and *mdh* (RL4439, 963 bp) genes, whereas the mutant strain OPS1012 was used for the non-phosphorylatable *manX* version with a single amino acid substitution at H9A. They were PCR amplified with Phusion™ High-Fidelity DNA Polymerase (New England Biolabs) using primers oxp0193/oxp0195 for *manX* and oxp0780/oxp0782 for *mdh*, and cloned into HindIII-KpnI linearised pOPINF vector using BD In-Fusion cloning (Clontech). The resulting plasmids pOPS0741, pOPS0114 and pOPS1067 for *mdh*, *manX*, and *manX* H9A, respectively, were confirmed by PCR sequencing with primers T7 and pOPINFrev.

**REFERENCES**

1. Sánchez-Cañizares C, Prell J, Pini F, Rutten P, Kraxner K, Wynands B, Karunakaran R, Poole PS. 2020. Global control of bacterial nitrogen and carbon metabolism by a PTS^Ntr^-regulated switch. Proc Natl Acad Sci U S A 117:10234–10245. <https://doi.org/10.1073/pnas.1917471117>.
2. Hanahan D. 1983. Studies on transformation of *Escherichia coli* with plasmids. J Mol Biol 166:557–580. <https://doi.org/10.1016/S0022-2836(83)80284-8>.
3. Johnston AWB, Beringer JE. 1975. Identification of the *Rhizobium* strains in pea root nodules using genetic markers. J Gen Microbiol 87:343–350. <https://doi.org/10.1099/00221287-87-2-343>.
4. Prell J, Mulley G, Haufe F, White JP, Williams A, Karunakaran R, Downie JA, Poole PS. 2012. The PTS^Ntr^ system globally regulates ATP-dependent transporters in *Rhizobium leguminosarum*. Mol Microbiol 84:117–129. <https://doi.org/10.1111/j.1365-2958.2012.08014.x>.
5. Terpolilli JJ, Masakapalli SK, Karunakaran R, Webb IU, Green R, Watmough NJ, Kruger NJ, Ratcliffe RG, Poole PS. Lipogenesis and Redox Balance in Nitrogen-Fixing Pea Bacteroids. J Bacteriol. 2016 Sep 22;198(20):2864-75. doi: 10.1128/JB.00451-16. PMID: 27501983; PMCID: PMC5038014.
6. Lodwig EM, Leonard M, Marroqui S, Wheeler TR, Findlay K, Downie JA, Poole PS. 2005. Role of polyhydroxybutyrate and glycogen as carbon storage compounds in pea and bean bacteroids. Mol Plant Microbe Interact 18:67–74.

<https://doi.org/10.1094/MPMI-18-0067>.

7. Untiet V, Karunakaran R, Krämer M, Poole P, Priefer U, Prell J. 2013. ABC transport is inactivated by the PTS^Ntr under potassium limitation in *Rhizobium leguminosarum* 3841. *PLoS One* 8(5):e64682. <https://doi.org/10.1371/journal.pone.0064682>
8. Knauf VC, Nester EW. 1982. Wide host range cloning vectors: a cosmid clone bank of an *Agrobacterium* Ti plasmid. Plasmid 8:45–54.

<https://doi.org/10.1016/0147-619X(82)90040-3>.

9. Figurski DH, Helinski DR. 1979. Replication of an origin-containing derivative of plasmid RK2 dependent on a plasmid function provided in trans. Proc Natl Acad Sci U S A 76:1648–1652. <https://doi.org/10.1073/pnas.76.4.1648>.
10. Schäfer A, Tauch A, Jäger W, Kalinowski J, Thierbach G, Pühler A. 1994. Small mobilizable multi-purpose cloning vectors derived from the *Escherichia coli* plasmids pK18 and pK19: selection of defined deletions in the chromosome of *Corynebacterium glutamicum*. Gene 145:69–73. <https://doi.org/10.1016/0378-1119(94)90324-7>.
11. Poole P, Reid C, East AK, Allaway D, Day M, Leonard M. 1999. Regulation of the *mdh-sucCDAB* operon in *Rhizobium leguminosarum*. FEMS Microbiol Lett 176:247–255. <https://doi.org/10.1016/S0378-1097(99)00243-8>.
12. Berrow NS, Alderton D, Sainsbury S, Nettleship J, Assenberg R, Rahman N, Stuart DI, Owens RJ. 2007. A versatile ligation-independent cloning method suitable for high-throughput expression screening applications. Nucleic Acids Res 35:e45–e45.
13. Keen NT, Tamaki S, Kobayashi D, Trollinger D. Improved broad-host-range plasmids for DNA cloning in gram-negative bacteria. Gene. 1988 Oct 15;70(1):191-7. doi:10.1016/0378-1119(88)90117-5. PMID: 2853689.
